# A New Data-Mining Method to Search for Behavioral Properties That Induce Alignment and Their Involvement in Social Learning in Medaka Fish (*Oryzias Latipes*)

**DOI:** 10.1371/journal.pone.0071685

**Published:** 2013-09-06

**Authors:** Takashi Ochiai, Yuji Suehiro, Katsuhiro Nishinari, Takeo Kubo, Hideaki Takeuchi

**Affiliations:** 1 Department of Biological Sciences, Graduate School of Science, The University of Tokyo, Tokyo, Japan; 2 Department of Physiology, Tokyo Women's Medical University School of Medicine, Tokyo, Japan; 3 Research Center for Advanced Science and Technology, The University of Tokyo, Tokyo, Japan; Utrecht University, Netherlands

## Abstract

**Background:**

Coordinated movement in social animal groups via social learning facilitates foraging activity. Few studies have examined the behavioral cause-and-effect between group members that mediates this social learning.

**Methodology/Principal Findings:**

We first established a behavioral paradigm for visual food learning using medaka fish and demonstrated that a single fish can learn to associate a visual cue with a food reward. Grouped medaka fish (6 fish) learn to respond to the visual cue more rapidly than a single fish, indicating that medaka fish undergo social learning. We then established a data-mining method based on Kullback-Leibler divergence (KLD) to search for candidate behaviors that induce alignment and found that high-speed movement of a focal fish tended to induce alignment of the other members locally and transiently under free-swimming conditions without presentation of a visual cue. The high-speed movement of the informed and trained fish during visual cue presentation appeared to facilitate the alignment of naïve fish in response to some visual cues, thereby mediating social learning. Compared with naïve fish, the informed fish had a higher tendency to induce alignment of other naïve fish under free-swimming conditions without visual cue presentation, suggesting the involvement of individual recognition in social learning.

**Conclusions/Significance:**

Behavioral cause-and-effect studies of the high-speed movement between fish group members will contribute to our understanding of the dynamics of social behaviors. The data-mining method used in the present study is a powerful method to search for candidates factors associated with inter-individual interactions using a dataset for time-series coordinate data of individuals.

## Introduction

Forming a group and living gregariously enable group members to more efficiently find food and escape predation [1]. Social animals utilize adaptive information associated with food or predators rapidly and economically via “social learning”, which is defined as learning that is influenced by observing or interacting with other members [2]. Social learning is observed in a variety of animal species, including colonial and noncolonial insects [3], [4], fish [5], birds [6], and mammals [7].

The key process in social learning is “information transfer” from informed members to other members [8]–[10]. The information transfer process has been studied extensively in social insects such as honeybees and ants. Honeybee workers perform waggle dancing to inform other workers in the hive of the distance and direction of a food source [11], [12]. Ants communicate with each other using pheromones as chemical signals [4], [13]. Some fish species that do not seem to have symbolic or chemical communication also undergo social learning. In guppies and golden shiners, untrained individuals (observers) that observe and learn from trained fish (demonstrators) can learn the route to a feeding area by shoaling with the demonstrator [14], [15]. In these species, a small number of demonstrators can lead a larger group to food, either through social facilitation of foraging movements or by eliciting coordinated behavior [5], [16]. Few studies, however, have examined the types of movement demonstrator fish use to induce alignment of the observer fish that facilitates social learning. To study this issue, we used small fish, medaka (*Oryzias latipes*), which exhibit prominent coordinated and cohesive movement [17]. Here, we first demonstrated that the formation of a group by medaka fish facilitates visual associative learning with food information.

In the present study, we used our newly established and versatile data-mining method that enabled us to search for candidate explanatory variables that may affect an objective variable using a large dataset to search for candidate behaviors that induce inter-individual interaction. The results of the data-mining using time-series coordinate data of individual medaka fish led us to hypothesize that high-speed motion induces the alignment movement of other fish. Finally, we examined this hypothesis in a behavioral paradigm for visual food learning and demonstrated that high-speed movements of the demonstrators facilitate social learning by eliciting alignment by the observers.

## Results

### Facilitation of learning acquisition in grouped medaka fish

**Figure 1:**
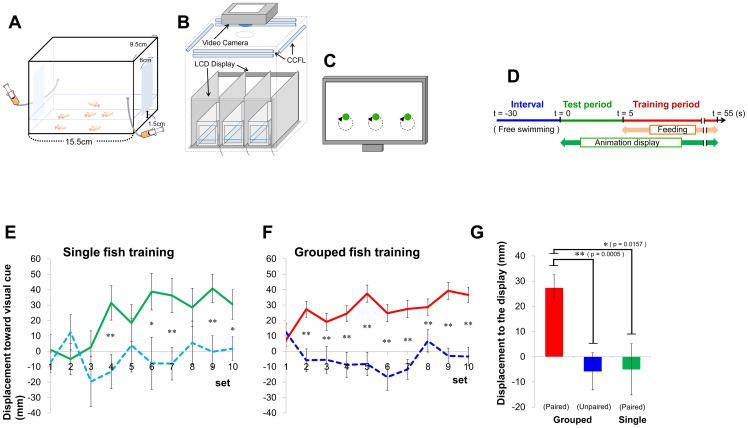
The novel behavioral assay to assess the training efficiency between grouped and solitary medaka fish. **(A) The test-training tank.** The tank comprises opaque walls with two transparent windows permitting the fish to see the LCD displays on each side of the tank. The two feed dispensers and two LCD displays are fixed at each side of the tank. **(B) Apparatus used for visual learning.** The whole apparatus is covered with a black box whose ceiling has a video camera that records the fish. Cold cathode fluorescent lamps were used for lighting. Three test-training tanks were placed in each apparatus. Brine shrimp (n = 120) were supplied as a reward for each training trial. **(C) Visual stimulus.** An animation (circular motion of a 2.5-cm diameter green circle) was presented for each tank. **(D) Experimental procedure for testing and training.** Each procedure comprised a test and training period. The inter-trial interval was 245 s. The control group was fed the same number of shrimp as the training group, but they were fed during the inter-trial interval. **(E-G) Quantitative analysis of fish motion in response to the visual presentation.** We measured how long the individual fish moved towards the display during the test period (5-s). We calculated the distance moved (Mean±s.e.m.) for each set, which comprised two successive training trials. The total number of the sets was 10 (20 trials). **(E) Single fish training.** The training group is represented by the green line (n = 16) and the control group is represented by the blue dashed line (n = 7). Statically significant differences were detected in the 4^th^, 6^th^, 7^th^, 9^th^, and 10^th^ training sets (t-test, p<0.05, see Table S1 for actual p values). **(F) Grouped fish training.** The training group is represented by the red line (12 groups, 72 individuals) and the control group is represented by the blue dashed line (6 groups, 36 individuals). Statistically significant differences were detected in all training sets except the 1^st^ set (t-test, p<0.01). **(G) Comparison between grouped and solitary fish.** We compared the distance moved of grouped and single fish training for the 2^nd^ training set. A significant difference was detected (Dunnett method, p<0.05). The paired group: visual cues were paired with food. The unpaired group: visual cues were not paired with food.

To determine whether medaka fish are able to learn to associate a visual cue with a food reward, we established a new behavioral paradigm. An animated movie was displayed as a visual cue using an LCD monitor (an LCD monitor was located on each side of the tank, Fig. 1). During a 5-s test period, the fish were exposed to the visual cue at one of the two sides of the tank (Fig. 1B). Following the test period, food was supplied to the side of the tank at which the visual cue was displayed (training period). To prevent fish from forming spatial memory based on the location of the food dispensers, we alternated the sides used for the test-training trials. We defined “one set” as two test-trainings, one trial at one side and the other trial at the other side.

First, we compared the learning acquisition between the paired group and unpaired group using a single fish. In the paired group, for which visual cues were paired with food, the visual cue was immediately followed by food supplied to the side of the tank displaying the visual cue. In the unpaired group, for which visual cues were not paired with food, the food was supplied during the interval (Fig. 1D) and the visual cue and food were not temporally associated. An unpaired group is generally used as a negative control in an associative learning paradigm. Figure 1E shows the learning acquisition of both groups in terms of displacement towards the visual cue during the test period (5 s). In the paired group, the displacement was significantly increased and reached a mean of ∼40 mm. In the unpaired group, the displacement did not increase. The difference in the displacement between the two groups was statistically significant within 4 training sets (7 and 8 trials). Thus, medaka fish can learn to associate visual cues with food in this behavioral paradigm.

To investigate the effect of group formation on learning acquisition in this paradigm, we performed the behavioral test using groups of six fish (Fig. 1F). The distance moved reached a similar level as when using a single fish in the paired group. A significant difference in the displacement was detected between the paired and unpaired groups within 2 training sets (3 and 4 trials). The displacement of the grouped fish during set 2 was significantly greater than that of the single fish (Fig. 1G), indicating that grouped medaka fish learn to respond to the visual cue more rapidly than single fish, suggesting that social learning provides a process whereby medaka more rapidly learn adaptive behavior.

### A novel data-mining method to search for explanatory variables that may affect an objective variable

**Figure 2:**
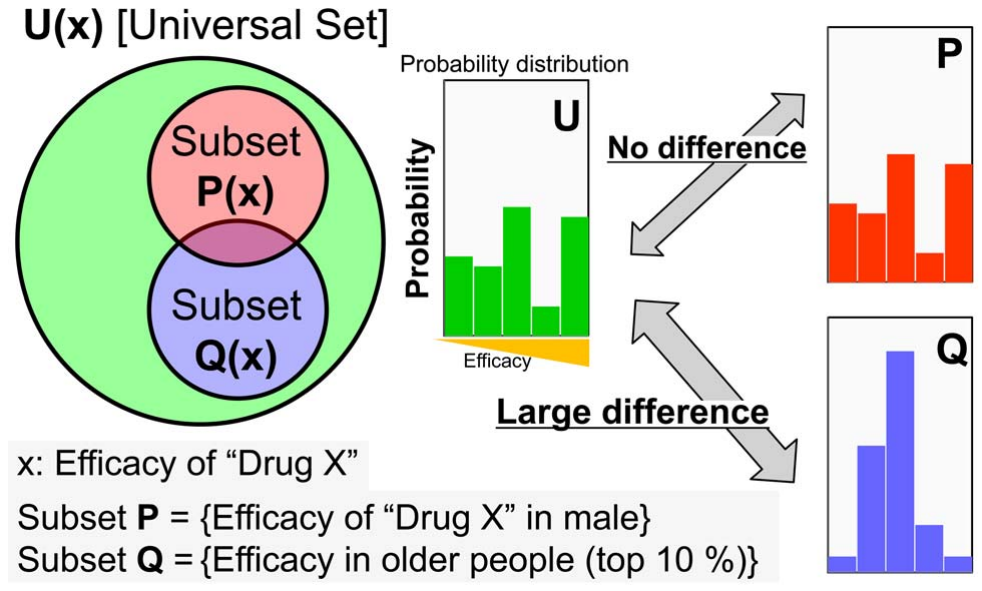
Outline of the calculation procedure used for the novel data-mining method. The purpose of data-mining is to search for candidate explanatory variables that may affect objective variables. The data-mining procedure comprises three steps; (1) Generation of **U** [the universal set] of an objective variable (x). This example shows that the objective variable is the efficacy of “Drug X”. (2) Generation of subsets from the universal set by the conditions that are defined based on explanatory variables. For example, subset **P(x)**, is generated by the condition “male” based on the explanatory variable “sex”, while subset **Q(x)** is generated by the condition “older people (top 10%)” based on the explanatory variable “age”. (3) Identification of candidate explanatory variables, by measurement and visualization of the difference in the probability distribution (histogram shape) of a subset from the universal set, **U(x)**. This example shows that the explanatory variable “age” may affect the drug efficacy, while “sex” does not.

We then searched for candidate behaviors that may affect alignment using time-series coordinate data of individual medaka fish. For this purpose, we established a versatile and simple data-mining method to search for candidate explanatory variables that may affect an objective variable using a large dataset. Here, we show a virtual experiment as an example (Fig. 2), whose purpose is to search for candidate explanatory variables that may affect the efficacy (x: an objective variable) of “Drug X”. A dataset of the drug efficacy in all the subjects was accompanied by explanatory variables such as sex, body weight, and age.

First, we define **U(x)** as the universal set whose elements are the drug efficacy (x) of all people. Next, we prepared subsets generated by conditions, which are defined based on the explanatory variables. For example, we can prepare two subsets (male and female) based on the explanatory variable “sex”. We can also prepare any number of subsets based on the explanatory variable “age”, such as a subset with age rank in the top 10% of all, subset **Q(x)**. If the probability distribution (histogram shape) of the subset of males, **P(x)**, was not different from that of **U(x)** [the universal set], the explanatory variable “sex” might not affect the drug efficacy (Fig. 2). If the probability distribution of the subset **Q(x)** was different, the condition “age rank in the top 10%” might affect the drug efficacy (Fig. 2). Thus, by assessing the difference in the probability distribution of a subset from that of **U(x)**, we can estimate the explanatory variables that may affect the drug efficacy.

Here, we applied Kullback-Leibler divergence (KLD) to assess differences in the probability distribution of subsets from the universal set. Given two datasets, **U** and **Q**, and the discrete probability distributions, ***U***(*i*) and ***Q***(*i*), the similarity between **U** and **Q** is given as the distance between ***U***(*i*) and ***Q***(*i*). KLD is not a true metric, and the word “distance” is used in a more general sense, but it is commonly used as a measure of the similarity between two distributions [18], [19]. The KLD of ***Q***(*i*) from ***U***(*i*) is defined as:
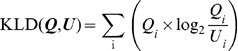
(1)


We chose the KLD from information theory, because KLD is applicable irrespective of the form of the probability distribution [20], [21], [22], [23]. We can measure the difference of the subset from the universal set, even if the probability distribution is not a unimodal distribution and/or it has considerable variation, as shown in Fig. 2. Further, a basic property of KLD is that it is not symmetric, i.e., in general.
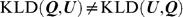
(2)


After preparing as many subsets as possible, which can be generated based on explanatory variables, subsets with high KLD scores can be screened. Visualization of the probability distribution (histogram shape) of subsets with high KLD scores enables us to make a reasonable assumption about the relation between explanatory and objective variables. The G-test is used to evaluate the statistical significance between **Q(x)** and **U(x)**. P-values were calculated from g-value and the chi-square distribution.
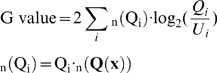
(3)


### Search for behaviors eliciting alignment movement using a data-mining method based on KLD

To search for behaviors that induce inter-individual interactions, we investigated the movement of individual fish within a group (6 fish) under free-swimming conditions without presenting a visual cue (Fig. 1D). It is noteworthy that, under this condition, fish interacted with each other without external visual signals. We measured the x-y coordinates of six individually marked fish (Fish i: i = 1∼6, individual identification number) for 30-s at 1-s intervals. The velocity vector of Fish i is defined as:
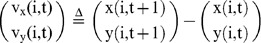
(4)


**Figure 3:**
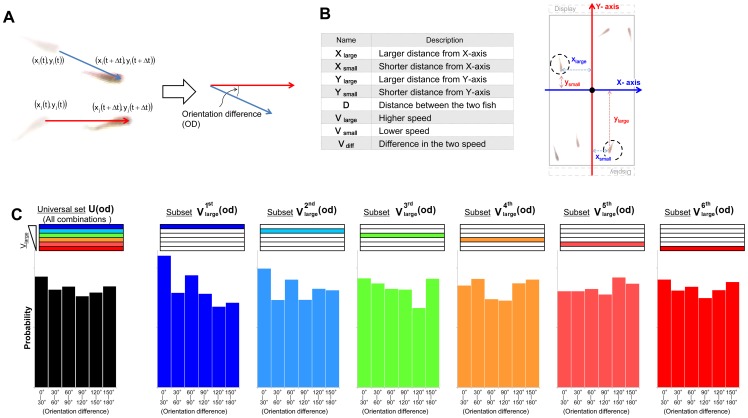
Searching for candidate explanatory variables that may affect inter-individual interactions. **(A) The difference in the orientation.** We focused on the difference between the orientations of a focal fish pair to measure the amount of inter-individual interaction. **(B) List of possible explanatory variables.** Five variables were concerned with position, and three variables were concerned with velocity. The variables concerned with position are shown in the schematic diagram. **(C) Visualization of the difference of probability distribution of six subsets generated based on V_large._** Each explanatory variable was stratified into 6 subsets (conditions) based on its score ranking. If the explanatory variable, V_large_ did not affect the OD, the probability distribution of the six subsets would be similar to that of the universal set. In contrast, if the condition (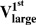 Group) affected the OD, the distribution would be different from that of the universal set.

To evaluate the inter-individual interactions of two fish, we measured the orientation difference (OD) between each combination of two fish (Fig. 3A). The OD_i,j,t_ of the two fish (Fish i and Fish j) is defined as:
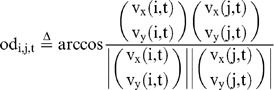
(5)


The OD range is [0, 180]. Alignment is represented by a low OD (0∼30°), while anti-alignment is represented by a high OD (150∼180°).

We then searched for candidate explanatory variables that may affect the OD using the data-mining method described in the previous section. Using the OD of two arbitrary individuals (t = 1∼30s, 1s interval) as an element, we prepared **U(od)** [the universal set]. **U(od)** comprised 4950 elements for the dyadic relationship (11 groups ×_6_C_2_×30 frames). The probabilities of all 6 intervals in **U(od)** were essentially the same (Fig. 3C, all combinations), indicating that the six fish did not continuously show significant inter-individual interactions as a group (6 fish).

We then searched for the conditions of fish position and movement in which a pair of fish from the 6-fish group showed transient and local inter-individual interactions. We defined eight explanatory variables concerning absolute and relative position and movement in a focal pair of fish (Fig. 3B).

X_ large_: Larger distance from X-axis.
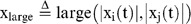
(6)X_ small_: Shorter distance from X-axis.
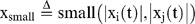
(7)Y_ large_: Larger distance from Y-axis.
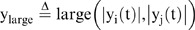
(8)


Y_ small_: Shorter distance from Y-axis.
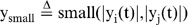
(9)


D: Distance between the two fish.
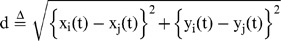
(10)


V_ large_: Higher speed.
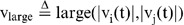
(11)


V_ small_: Lower speed.
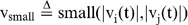
(12)


V_diff_: Difference of the two speeds.
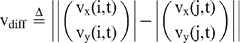
(13)


Based on time-series data (t = −30∼0 s at 1-s intervals) of x-y coordinates of Fish i and Fish j, we calculated the eight explanatory variables and prepared datasets of OD accompanied by the eight explanatory variables.
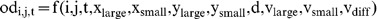
(14)


**Figure 4:**
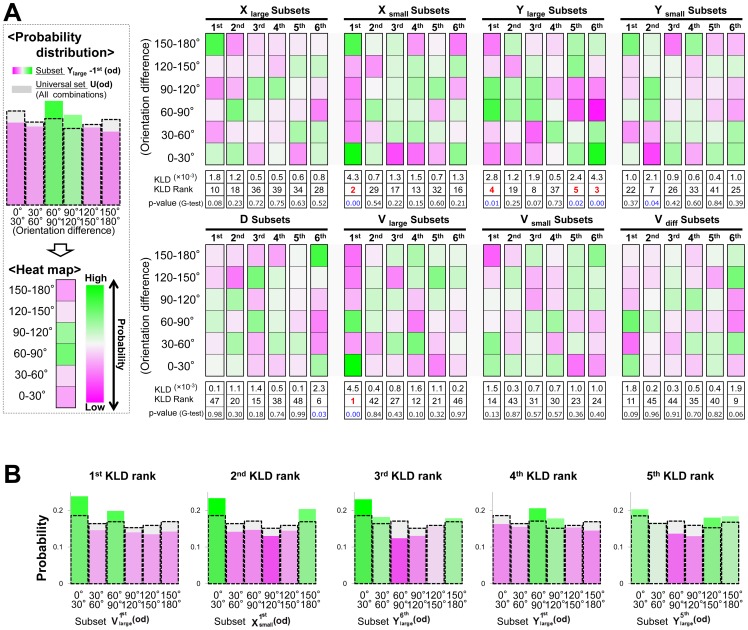
Top five subsets showing high-scored KLD. **(A) Measurement of the difference of subsets from the universal set.** Here we represent the probability distribution using a heat map. The OD histogram-intervals with a higher proportion than that of all combinations are shown in green, while those with a lower proportion are shown in magenta. The KLD value and KLD rank are indicated below each column. Below them, the G-test p-values are shown. High KLD values indicate low similarity and we selected the top five. **(B) Visualization of the probability distributions of the top five subsets exhibiting high-scored KLD.**

To search for candidate explanatory variables that affect the OD, we divided **U(od)** into six subsets based on the magnitude of each explanatory variable (score ranking) and measured the difference of subsets from the universal set using the KLD (Fig. 4). If the explanatory variables did not affect the OD, the probability distribution of all six subsets would be similar to **U(od)** (Fig. 3). In the present study, we measured the difference of a total of 48 subsets (6 subsets ×8 explanatory variables; Fig. 4).

The probability distribution of the subset with the 1^st^ KLD rank was skewed left (Figs. 3C and 4B), and was defined by the highest magnitude of V_large_ (subset 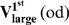). In contrast, the probability distribution of the subset that was defined by the lowest magnitude of V_large_ (subset 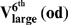) was almost flat with low KLD rank (46^th^) (Figs. 3C and 4A). The G-value revealed a significant difference in the subset 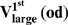 (p<0.01), but not in the five lower subsets (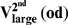 **∼** 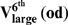 **)**, indicating that high-speed movement of either fish tended to affect alignment of the other fish in a focal pair.

We also performed the same analysis based on other subdivisions (Figs. S2 and S3). In cases of 3 subdivisions, the KLD ranks of subset 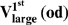 was 4^th^ of 12 subsets and the G-test revealed a significant difference (p<0.01) in the subset 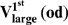, but not in the two lower subsets (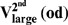 **, and** 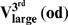 **)**. In cases of 9 subdivisions, the KLD ranks of subset 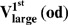 was 6^th^ of 72 subsets, and the G-test revealed a significant difference (p<0.01) in the subset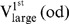, but not in the eight lower subsets 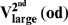 **-** 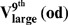. In addition, we examined other interval widths (Fig. S2). In cases of 4 and 9 OD subdivisions (45^o^ and 20^o^ interval widths, respectively), the KLD ranks of subset 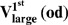 were 3^rd^ and 4^th^, respectively, and the G-test revealed a significant difference (p<0.01; Fig. S3). These findings suggested that changing the number of subdivisions does not strongly affect the results of the present data-mining method.

Here the skewed left shape (Figs. 3C and 4B) of 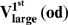 led us to hypothesize that the focal pair tends to show alignment movements under conditions in which either of the focal pair fish moves with a relatively high speed (among the top one-sixth), because the probability is relatively high within these intervals [0°, 30°]. The probability distribution (histogram shapes) of the subsets with the highest, 2^nd^, 3^rd^, 4^th^, and 5^th^ rank KLD were not skewed to either the left or the right (Fig. 4B). As the four subsets were defined by the magnitude of Y_large_, and X_small_, which represented the location of the fish in the tank, the biased frequency distribution of OD might be due to the spatial restriction in the tank rather than inter-individual interactions (See Fig. S1 for details).

### High-speed access of learned fish to the food source in response to the visual cue

**Figure 5:**
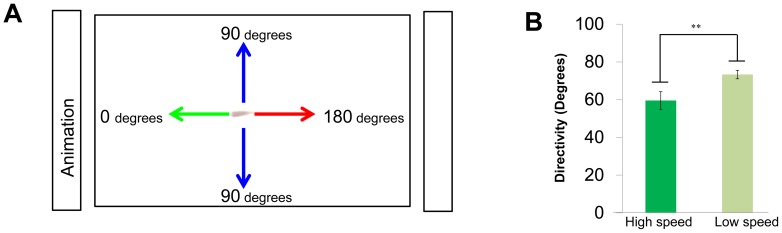
The difference in the directivity orientation between high speed and low speed. **(A) Definition of directivity.** The directivity value was 0**(B) Difference in the directivity in the test period of solitary learning.** From the 6^th^ to the 10^th^ test period of solitary learning, the mean value of the directivity angle at high speed was 59.6 degrees (N = 160), while that at low speed was significantly higher at 73.3 degrees (N = 640; t-test, p = 0.0044).

The result of the previous data-mining prompted us to examine whether the alignment elicited by high-speed movement facilitated access to a food source in response to a visual cue. First, we examined whether trained fish moved at a high speed in response to a visual cue. We defined trained fish as those that received more than 5 training sets (>10 trials) in the single-fish training paradigm in the previous experiments (Fig. 1E) and analyzed the movement of the trained fish during the test period. Then we defined the directivity to the display. When the fish moved forward toward the visual cue, directivity was defined as 0° (Fig. 5A). When they moved backward, directivity was defined as 180°. We compared the directivity of fish moving at high and low speeds (in the top 20% and the lower 80% of each individual, respectively). Directivity was significantly different at high-speed (59.3±4.7°) and low-speed (73.3±2.3°, mean±s.e.m.; t-test, p = 0.0044), indicating that the trained fish rapidly moved toward the visual cue (Fig. 5B).

### Demonstrator-elicited approach behavior to the visual cue in observers

**Figure 6:**
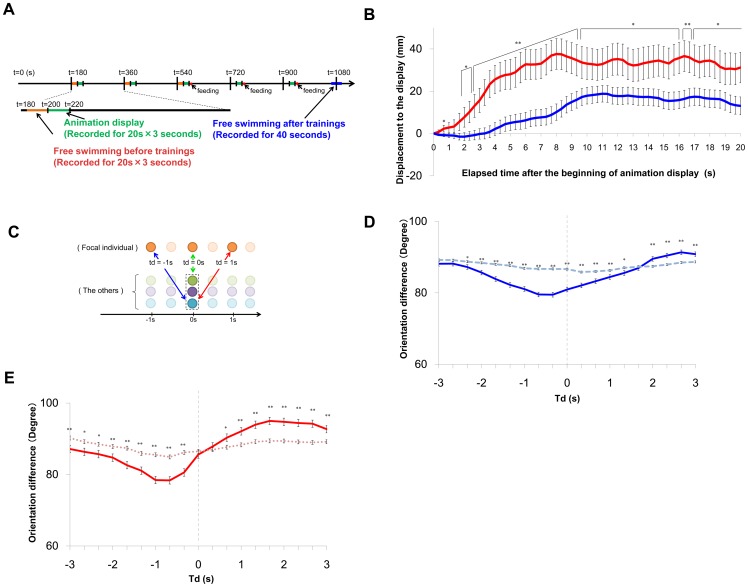
Measurement of the orientation differences between focal fish and the others with a time difference. **(A) Time table of Experiment 2.** In this experiment, we used 4 fish per group: one trained individual and three untrained ones. The trained fish had learned to move towards the visual cue. **(B) Change in the distance moved to the visual cue.** During the animation display before training, we measured the mean movement from the individuals to the display at 1/3-s intervals. The red line indicates the mean movement of the demonstrators, which peaked at 37.6 mm at 8.0 s. The blue line indicates the mean distance of the observers, which peaked at 18.8 mm at 11.0 s. Bars indicate s.e.m. After 2 s, a statistically significant difference was detected (t-test, p<0.05, see Table S1 for actual p values). **(C, D) The orientation differences (OD) when the phase was shifted during free-swimming before training. (C) Definition of time difference (Td).** Td refers to the time difference between the focal fish and the other fish. **(D) The orientation difference (OD) of all the individuals at every Td.** The orientation difference 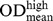 when the focal fish swam at high speed is indicated as the solid blue line, and the orientation difference 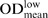 when the focal fish swam at low speed is indicated as the dashed blue line [see equation (17)]. The 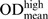 at high speed fell from −7/3s to 4/3s, but exceeded 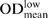 from 2s to 3s (t-test, P<0.01, Bar; S.E.). Statistically significant differences are indicated by asterisks (t-test, *: p<0.05, ** : p<0.01). **(E) The orientation differences versus Td during the visual cue.** The focal fish are the demonstrators. The solid red line indicates the 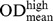 when demonstrators swam at high speed, and the dashed red line indicates the 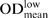 when the demonstrators swam at low speed. Statistically significant differences are indicated by asterisks (t-test, * : p<0.05, ** : p<0.01).

We then evaluated whether the response of the trained fish (demonstrators) to the visual cue elicited approach behavior by the observers (untrained fish) to the same visual cue without supplying the food reinforcement. Here we prepared a 4-fish group comprising one demonstrator and three observers and analyzed the time-series data (200∼220, 380∼400, and 560∼580 s at 1/3-s intervals) of x-y coordinates of the four individually marked individuals (Fish i: i = 1∼4, individual identification number) in response to the visual cue (Fig. 6A). Displacement towards the visual cue of the demonstrator fish peaked at 37.6±7.2 mm (mean±s.e.m.) at 8.0 s after presentation of the visual cue, while that of the three observers was 18.8±3.9 mm at t = 11.0 s (Fig. 6B). This finding suggests that the response of the demonstrator to the visual cue elicits approach behavior by the observers, suggesting that that the demonstrator's behavior induces the observers to approach the food source during the test and training period.

### Behavioral cause-and-effect of rapid response between group members

We then evaluated whether a focal fish moving at high speed induces the alignment of other members with a time delay during free-swimming without presentation of a visual cue (Fig. 6C). We calculated the mean OD between the focal fish (Fish i) and the other three fish (Fish j_1_∼j_3_) with a time difference (Td), based on time-series data (t = 180∼200, 360∼380, and 540∼560 at 1/3-s intervals, Fig. 6A) of x-y coordinates of Fish i (t+td) and Fish j_1_∼j_3_ (t). The OD with a time difference between two fish, Fish i (t+td) and Fish j_k_ (t) is defined as:
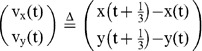
(15)

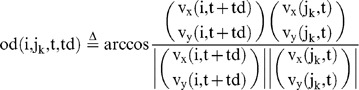
(16)


The mean OD between the focal fish (Fish i) and the other three fish (Fish j_1_∼j_3_) with a time difference (Td) is defined as:
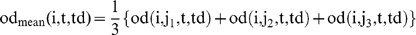
(17)


To examine the effect of fish speed on the OD_mean_ , we prepared two subsets based on fish speed.
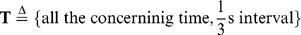
(18)

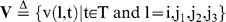
(19)

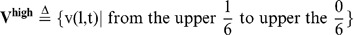
(20)

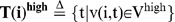
(21)

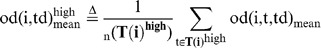
(22)

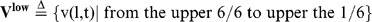
(23)

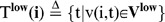
(24)

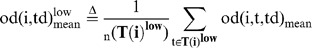
(25)


Figure 6D shows the 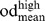 and 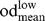 between the focal fish and other three fish with a time difference (Td). As expected, the 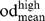
(the focal fish swam at high speed; solid lines) decreased with a time delay, with a minimum value (76.8±0.68°, mean±s.e.m., N = 34) at td = −2/3 s, indicating that the decrease in the 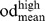 followed the high-speed movement of the focal fish. In contrast, the 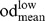 (the focal fish swam at low speed; dashed lines) was approximately 90° at any time difference, indicating that there was no interaction between group members when the focal fish swam at low speed. Thus, high-speed movement of an individual in a group tended to elicit alignment of the other members with a 1/3- to 1-s delay.

### Elicitation of alignment in observers by high-speed movement of the demonstrator during visual cue presentation

We then examined whether the observers exhibited alignment when the demonstrators exhibit high-speed movement during the visual cue presentation. Figure 6E shows the 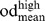 and between the demonstrator (Fish i) and observers (Fish j_1_∼j_3_) with the time difference (See equations 17,30,31). The time difference (Td) is the time lag of Fish i from Fish j_1_∼j_3_ (as a standard). The 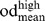 (the demonstrator, as the focal fish, swam at high speed; solid lines), reached a minimum (78.4°±1.0°, mean±s.e.m., N = 34) at td = −1 s. In contrast, (the demonstrator, as the focal fish, swam at low speed; dashed lines) was approximately 90° at any time difference. These findings strongly suggested that the observers tended to exhibit alignment in response to the high-speed movement of the demonstrator with a 1/3- to 4/3-s delay during presentation of the visual cue.

### Possible involvement of individual recognition in social learning

**Figure 7:**
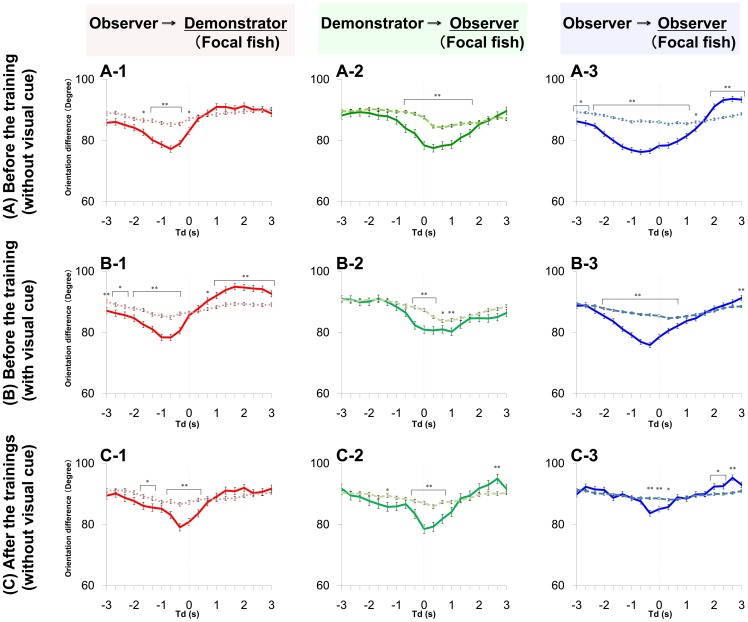
Orientation differences with time difference. The orientation difference 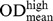 when the focal fish swam at high speed is indicated as a solid line, the orientation difference 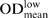 when one fish swam at a low speed is indicated as a dashed line. (**A**) **During free-swimming before training.** (**A-1**) **The** 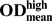 **and** 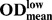 **between the one demonstrator and three observers when the focal fish were demonstrators.** Corresponding equations are (37) and (41). (**A-2**) **The** 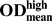 **and** 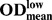 **between three observers and one demonstrator when the focal fish were observers.** Corresponding equations are (47) and (50)**.** (**A-3**) **The** 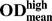 **and** 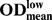 **between one observer and the other two observers, when the focal fish were observers.** Corresponding equations are (57) and (60)**.** (**B**) **During the visual cue presentation before training.** (**C**) **During free-swimming after training.** Actual p values are shown in Table S1.

**Figure 8:**
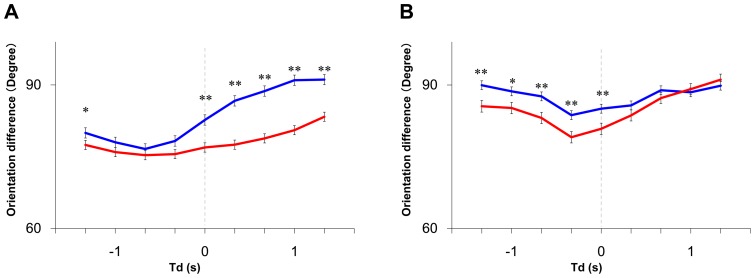
Comparison of the orientation difference of the demonstrator and the observers. We compared the 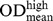 between a demonstrator and an observer with the 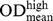 between an observer and another observer. (**A**) **During free-swimming before training.** The blue line indicates the orientation difference 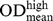 between the observer at high speed and the other two observers. Corresponding equations is (57). The orientation difference was smaller than the red line indicating the difference 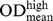 between the demonstrator at high speed and the three observers. Corresponding equation is (38). Statistically significant differences are indicated by asterisks (t-test, * : p<0.05, ** : p<0.01, see Table S1 for actual p value.). **(B) During free-swimming after training.** The red line was smaller than the blue line, and statistically significant differences are indicated by asterisks (t-test, * : p<0.05, ** : p<0.01).

Given that medaka fish could recognize a demonstrator (trained fish) in the group, naïve fish that exhibited alignment movement with a demonstrator would increase their own foraging success through social learning about food sources. Therefore, we examined whether the high-speed movement of the demonstrator is more likely to induce alignment movement of other observers than the movement of an observer. Here we calculated the 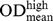 during free-swimming without the visual cue before (t = 180∼200, 360∼380, and 540∼560 s) and after three trainings (t = 1060∼1100 s). As the focal fish, we used a demonstrator or a randomly selected observer (See Equations 38 and 57, respectively). It is noteworthy that, under this condition, fish interacted with each other without external visual signals (Fig. 7A and C). Before training, irrespective of whether the focal fish was the demonstrator or observer, the 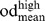 decreased with a time delay and had minimum values (76.6± 1.1° and 75.3± 0.96°, respectively, mean±s.e.m., N = 31, p = 0.3667) at td = −2/3 s (Fig. 8A). The difference between the two minimum values of the 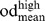 was not significant (Fig. 8A). The 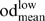 in the demonstrator and observer were approximately 90° at any time difference (Fig. 7A). The similar tendencies were also detected during the visual cue presentation before trainings (Fig. 7B). In contrast, during free swimming after the three training trials, there was significant difference depending on whether the focal fish was the demonstrator or observer (Fig. 7C). The 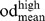 of the demonstrator were significantly lower than those of the observer at any negative time difference (td = 0∼−3/4 s; Fig. 8B). The minimum values (79.1± 1.2° and 83.7± 0.93°, respectively, mean±s.e.m., N = 31, p = 0.0023) at td = −1/3 s were significantly different. The 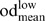 of both the demonstrator and observer were approximately 90° at any time difference (Fig. 7C). Our findings suggested that naïve fish (observer) exhibited diminished ability to induce alignment in the other observer fish during the training trials whereas the demonstrator had a relatively high tendency to induce alignment after three trainings.

## Discussion

Here we demonstrated that grouping in medaka fish facilitated learning of visual information associated with a food source, and that naïve “observer” medaka fish would follow trained “demonstrator” fish. Generally, fish in large groups forage more efficiently than those in small groups via socially transmitted information, which has been demonstrated in goldfish (*Carassius auratus auratus*)[24], bluntnose minnows (*Pimephales notatus*) [25], Alaska pollack (*Theragra chalcogramma*) [26], sticklebacks (*Gasterosteus aculeatus*) [27], [28], golden shiners (*Notemigonus crysoleucas*), and guppies (*Poecilia reticulata*) [10], [14], [29], [30]. Theoretical studies revealed that group coordinated dynamics can be explained by interactions between individuals, such as collision avoidance and alignment among group members [31]. The development of methods to determine the behavioral cause-and-effect between group members that mediates social learning, however, has been an obstacle [32]. In a medaka fish group under free-swimming conditions without an external stimulus, we could not detect any tendency to maintain the nearest neighbor distance (data not shown) or to exhibit alignment movement as a group, which is consistent with our previous studies showing no significant inter-individual interaction between a pair of medaka fish under free-swimming conditions [17]. Here, we demonstrated that the high-speed movement of a focal fish tends to induce alignment of the group members locally and transiently. This behavioral property can mediate a process of social learning called “observational conditioning”, which refers to the process in which “the response of a demonstrator to a stimulus elicits a matching response on the part of an observer, that simultaneously perceives the original stimulus, and effectively learns that the response is an appropriate response to it” [33], [34]. The behavioral cause-and-effect based on the high-speed movement between group members will help us to understand the dynamics of collective behaviors.

Our findings also suggest that individual recognition among a group facilitates social learning. This raises the possibility that an observer could recognize other observers as uninformed fish and choose not to respond to their movement, as the observer obtained no food by following the other observers. Theoretical models of social learning suggest that the formation of a group where social interactions occur at random can facilitate the learning process. Reader & Laland (1999), however, demonstrated that sex, age, and hunger level strongly influence foraging performance in the guppy [35], implying “directed social learning”, where transmitted information is restricted to a subset of individuals [36]. Our findings suggested the existence of “directed social learning” in medaka fish, where information is efficiently transmitted from trained fish to naive fish by individual recognition.

Although advances in video-tracking and GPS systems allow for automatic location of one member of a group of animals [37], [38], we had no standard data-mining method to search for unknown behavioral rules in a hypothesis-independent manner based on the times-series coordinates of individuals. In the present study, we established a novel data-mining method based on the KLD. We first used it to analyze inter-individual interactions in a hypothesis-independent manner using the times-series coordinates of individuals. Although we used dynamics and positional information of individuals as explanatory variables in the present study, other quantitative indices such as physiologic state, body size, and age, as well as qualitative indices such as sex, rearing conditions, familiarity, and strain, can be used as explanatory variables. Generation of some subsets based on an explanatory variable of interest allows investigators to estimate whether the explanatory variable is related to the inter-individual interaction by comparing the shapes of the probability distribution of subsets based on the KLD. Combining the present data-mining method and recent advances in data tracking and GPS systems will allow us to find unknown behavioral rules in animal groups.

## Materials and Methods

### Ethics statement

The work in this paper was conducted using protocols approved by the Animal Care and Use Committee of the University of Tokyo (permit number: 12–07). All surgery was performed under cold anesthesia, and all efforts were made to minimize suffering.

### Animals

Medaka (Himedaka, *Oryzias latipes*) were obtained from a local pet shop and females (20–25 mm body length) were selected for use in the study. The fish were housed in a plastic rearing tank (12×13×19 cm) maintained at a temperature of 28°C under a 14 h:10 h light:dark cycle.

### Associative visual learning

During the experiment, the fish were transferred into the test-training tank (15.5×9.5 cm×10.8 cm, water depth 2.5 cm) with covered opaque walls and two transparent windows (6×7 cm) to allow the fish to see the LCD displays placed at each side of the tank (Fig. 1A). The feed dispensers comprised a syringe and silicon tube fixed to the sides of the tank. The feed dispenser and two LCD displays (RDT153LM, Mitsubishi) were set beside the test-training tank. Movement of the fish was recorded from above the tank in high-definition movies using a digital single-lens reflex camera (EOS T4i and EF50mm F1.8 II, Canon). Cold cathode fluorescent lamps were placed over the tanks as light sources. Three test-training tanks were used for each set of LCD displays.

### Visual associative learning using only naïve fish

For grouped training, we labeled the right or left back of the fish with silicon ink, which allowed us to discriminate among individuals using a recorded movie. The silicon ink was injected under the skin of ice-anesthetized fish using a syringe (TERMO SV-S19EL, SS-02SZ). Using three colors, we labeled the six fish of each group as follows: right-pink, left-pink, right-blue, left-blue, right-green, and left-green. During the 3 days after the injection, groups of 3 to 4 fish were kept in a large opaque cup. Prior to the day before the training day, the fish were single-housed in opaque cups. For grouped control, single training, and single control fish, a needle was penetrated into the back of the fish but no silicon ink was injected. The other manipulations were the as same as for the grouped training.

As the training-test procedure for grouped and single training, each fish group (6 fish) was transferred from the rearing tank into the training tank, and the training and test procedure was started 15 min later. We repeated the test and training procedure 20 times. Each procedure comprised both test and training periods, and the interval between each procedure was 245 s. At the beginning of the procedure, we presented the animation (visual cue), i.e., a 2-cm green circle moved in a 2.5-cm diameter circle at 60-rpm, on the display on one side of the tank. After a 5-s interval, 120 brine shrimp were placed into the tank through the silicon tube next to the LCD display showing the visual cue for a period of 55 s. The positions of the visual cue display and food presentations were alternated from one side to the other. For grouped control and single control fish, we fed the fish 120 brine shrimp during the inter-trial interval and not during the visual cue display period. All manipulations were the same as for grouped training. The other manipulations were the same as the grouped training.

### Data collection

We measured the distance each individual fish traveled toward the display during each test period. To analyze fish movement during free swimming, we converted the video images into image sequences using QuickTime and manually obtained time series (1 per second) position data of each fish using a touch panel PC and Image J. We entered the actual path by manually following the track of the animal, because there is no methodology currently available that allows for the automatic detection of the location of every individual in a group of freely swimming fish from a video source. Due to a technical error, data for only 11 of the 12 training groups were analyzed.

### A data-mining method based on KLD

First, we generated the **U(od)** ,the universal set of an orientation difference (OD). The OD can be described as the image of explanatory variables:
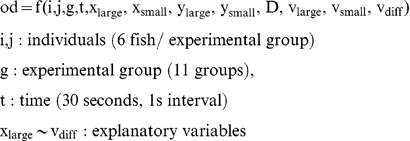
(26)


Next we generated six subsets from **U(od)** based on score-ranking of each explanatory variable. We made six groups of each explanatory variable and then generated the six OD subsets.



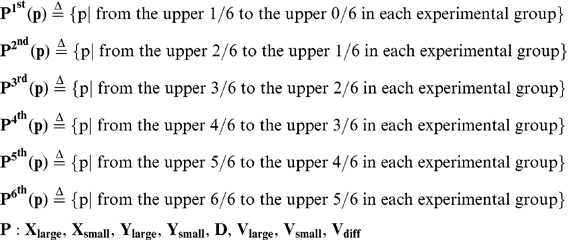
(27)

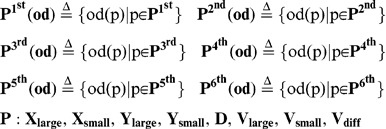
(28)We generated 48 OD subsets (8 explanatory variables pla) and created discrete probability distributions. The discrete probability distributions had 6 intervals [0°, 30°], [30°, 60°], [60°, 90°], [90°, 120°], [120°, 150°], and [150°, 180°]. Finally, we measured the difference of the distribution of subsets from that of the universal set using the KLD.



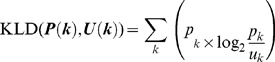

*k* : [0°, 30°], [30°, 60°], [60°, 90°], [90°, 120°], [120°, 150°], and [150°, 180°].
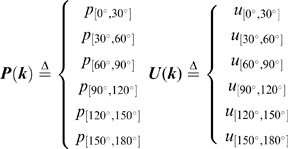


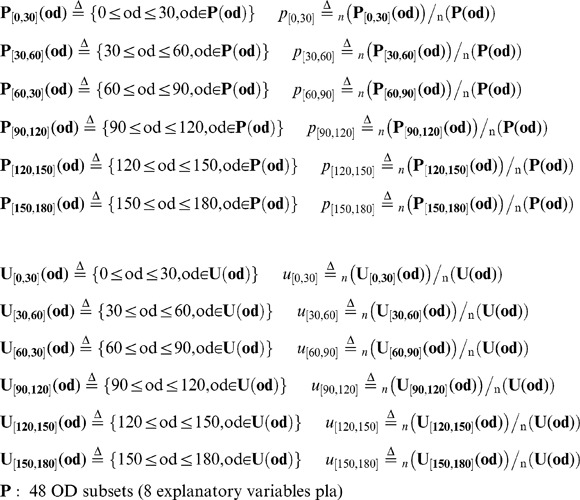
(29)


In addition to the discrete probability distributions with 6 intervals, we also generated probably distributions with 4 or 9 intervals (See Fig. S2 for details).

### Measurement of the difference of the directivity angle between high speed and low speed

The index of the directivity angle was defined as the angle between the direction of movement of a focal fish and the angle perpendicular to the display. The index of directivity was 0 if the fish moved directly towards the visual cue, and it was 180 if the fish moved backwards. We determined the high speed-movement for each training fish in each set. Each set comprised 10 periods (1 per second), and high speed was defined as the 2 periods with the fastest movement out of the 10 periods. Low speed was defined as the speed of movement in the remaining 8 periods. We calculated the mean directivity for high-speed movement and that for low-speed movement.

### Visual associative learning using a four–fish group (1 demonstrator and 3 observers)

To generate and identify a demonstrator, we labeled the fish with silicon ink as described above. During the 3 days after the injection, each fish was kept in a large opaque cup. We then trained each fish separately to learn the association between the visual cue and food reward for 3 days. For the training, the inter-trial interval was 195 s and each fish received 14 training trials. The other manipulations were the same as in the first experiment. We trained 24 fish and selected 12 fish that demonstrated approach behavior at least 6 times in response to the visual cue as demonstrators. Observers were kept singly in a large cup and fed the same amount of food as the trained fish.

The day before the group training trials began, we trained the trained fish at least twice with 14 trials and a 195-s inter-trial interval. The observers were kept singly in a large cup and fed as much food as the demonstrators.

The next day, the trained fish and untrained fish were placed in the training-test tank. To select naïve fish as observers, we presented the visual cue 6 times for 20 s with 160-s intervals. We selected the fish that did not shift to the visual cue based on observation of the demonstrator fish. Demonstrator fish were selected based on the size of the untrained fish to make the size of all four individuals in a group as uniform as possible. Before putting the four fish into the training-test tank, we trained the trained fish 4 times with a 195-s inter-trial interval. The one demonstrator fish and three observer fish were placed into the training-test tank together. At 180 s after the fish were placed into the tank, the visual cue was presented twice for 20 s with a 160-s inter-trial interval. Next, the visual cue was presented for 20 s, and then 120 brine shrimp were immediately supplied to the tank. This procedure was repeated three times with a 160-s inter-trial interval (Fig. 6A).

### Calculation procedure of the mean of the orientation difference (OD) when the focus fish is changed

(A) The OD among the four fish. The time difference (Td) is the time lag of Fish i from Fish j_1_∼j_3_ (as a standard). After the calculation of metrics (15)–(21),(23)–(24), we calculated the two metrics, 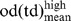 and 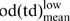.
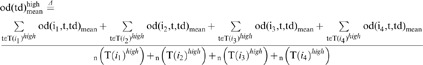
(30)

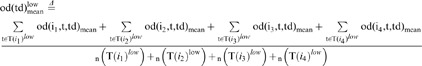
(31)(B) The focal fish (Fish i) is the demonstrator and the other three fish are Fish j_1_∼j_3_. The time difference (Td) is the time lag of Fish i from Fish j_1_∼j_3_ (as a standard). We calculated the two metrics, 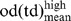 and 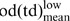 .
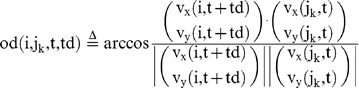
(32)

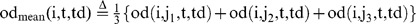
(33)

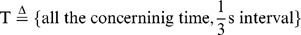
(34)

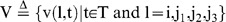
(35)

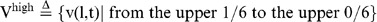
(36)

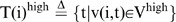
(37)

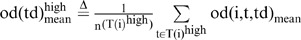
(38)

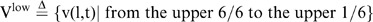
(39)

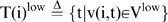
(40)

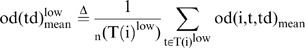
(41)(C) The focal fish is the observer (either of Fish i_1_∼i_3_) and the other fish is the demonstrator Fish j. The time difference (Td) is the time lag of Fish i_k_ from Fish j (as a standard). We calculated the two metrics, 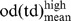 and 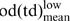 .
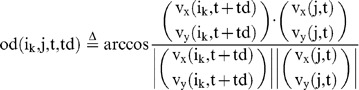
(42)

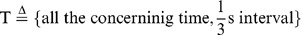
(43)

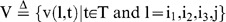
(44)

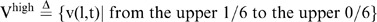
(45)

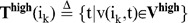
(46)

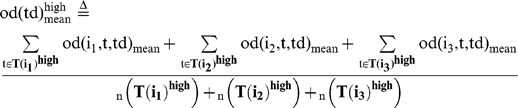
(47)

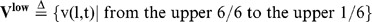
(48)

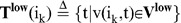
(49)

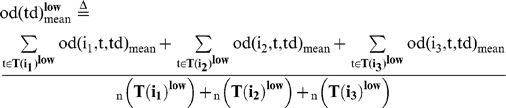
(50)(D) The focal fish is an observer (either of Fish i_1_∼i_3_) and the other two observers Fish j_1_∼j_2_. The time difference (Td) is the time lag of Fish i_k_ from Fish j_i_ (as a standard). We calculated the two metrics, 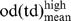 and 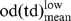 .
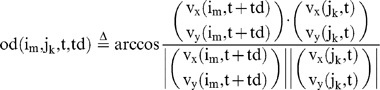
(51)

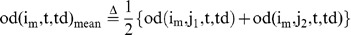
(52)

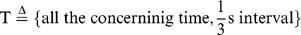
(53)

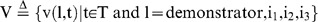
(54)

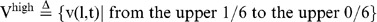
(55)

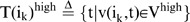
(56)

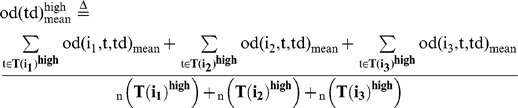
(57)

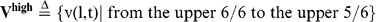
(58)

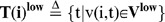
(59)

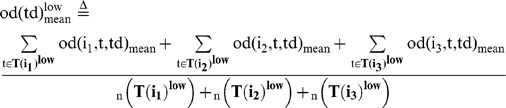
(60)


## Supporting Information

Figure S1
**Subsets with the five highest KLD scores. (A)** Subset 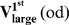: If either of the two fish were swimming at high speed, they tended to swim in the same direction (0–90 degree angle). **(B)** Subset 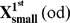: If two fish were near the long axis of the wall, they tended to move in the same or opposite direction, and the probability of a 0–30 degree angle was higher than that for a 150–180 degree angle. **(C)** Subset 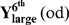 **and** Subset 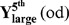: If two fish were far from the short axis of the wall, they tended to move in the same or opposite direction, and the probability of a 0–30 degree angle was higher than that for a 150–180 degree angle. **(D)** Subset 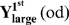: If either of the two fish located near the short axis of the wall, their orientation difference tended to be 90 degrees.(TIF)

Figure S2 **KLD and p value based on other subdivisions (3 and 9).** We represent the probability distribution using a heat map. The OD histogram-intervals with a higher proportion than that of all combinations are shown in green, while those with a lower proportion are shown in magenta. The KLD value and KLD rank are indicated below each column. Below them, p values for the G-test are shown. **(a) 3 subsets division based 8 explanatory variables. (b) 9 subsets division based 8 explanatory variables.**
(TIF)

Figure S3 **KLD and p value when the discrete probability distribution has 4 or 9 intervals. (A) 9-intervals.** We represent the probability distribution using a heat map. The OD distribution has 9 intervals; [0°–20°], [20°–40°], [40°–60°], [60°–80°], [80°–100°], [100°–120°], [120°–140°], [140°–160°], and [160°–180°]. The OD histogram-intervals with a higher proportion than that of all combinations are shown in green, while those with a lower proportion are shown in magenta. The KLD value and KLD rank are indicated below each column. Below them, p values for the G-test are shown. **(B) 4-intervals.** The probability distribution has 4 intervals; [0°–45°], [45°–90°], [90°–135°], and [135°–180°]. **(C) Comparison of p value on G-test.** The p values under 0.05 are colored blue.
(TIF)

Table S1 **Summary for p values in** **Figs. 1** **,** **6** **,** **7** **, and** **8** **.** A p value less than 0.01 is indicated in red, and that less than 0.05 is indicated in blue.
(TIF)

## Data availability

The raw data are available to readers by contacting the corresponding author.

## Permit

The work in this paper was conducted using protocols approved by the Animal Care and Use Committee of the University of Tokyo.
