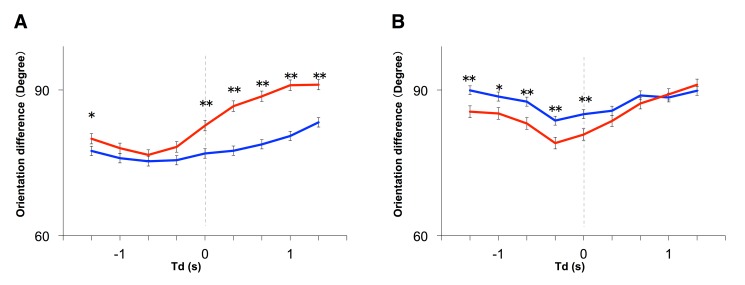# Correction: A New Data-Mining Method to Search for Behavioral Properties That Induce Alignment and Their Involvement in Social Learning in Medaka Fish (*Oryzias Latipes*)

**DOI:** 10.1371/annotation/a895afc8-446c-4e3e-a71b-52f2baa9df10

**Published:** 2013-11-13

**Authors:** Takashi Ochiai, Yuji Suehiro, Katsuhiro Nishinari, Takeo Kubo, Hideaki Takeuchi

The line colors were switched in Figure 8A. Please see the corrected Figure 8 here: 

**Figure:**