# Supplementary figures and images for: A New Data-Mining Method to Search for Behavioral Properties That Induce Alignment and Their Involvement in Social Learning in Medaka Fish (Oryzias Latipes)
Source: PLoS One. 2013 Sep 6;8(9):e71685. doi: 10.1371/journal.pone.0071685 (PMC3765494; doi:10.1371/journal.pone.0071685)

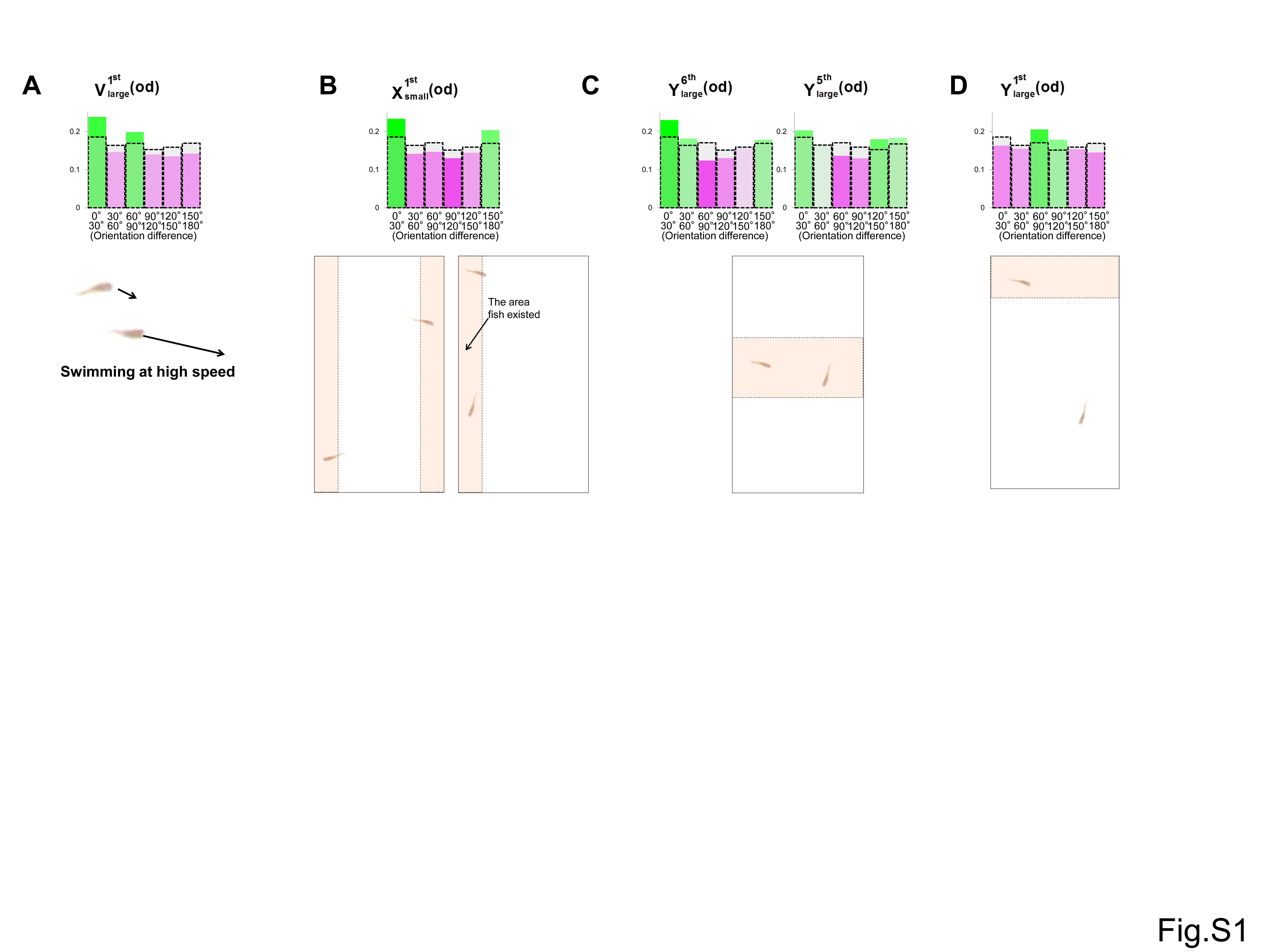

Supplement: Figure S1 — Subsets with the five highest KLD scores. (A) Subset : If either of the two fish were swimming at high speed, they tended to swim in the same direction (0–90 degree angle). (B) Subset : If two fish were near the long axis of the wall, they tended to move in the same or opposite direction, and the probability of a 0–30 degree angle was higher than that for a 150–180 degree angle. (C) Subset and Subset : If two fish were far from the short axis of the wall, they tended to move in the same or opposite direction, and the probability of a 0–30 degree angle was higher than that for a 150–180 degree angle. (D) Subset : If either of the two fish located near the short axis of the wall, their orientation difference tended to be 90 degrees. (TIF) [file pone.0071685.s001.tif]

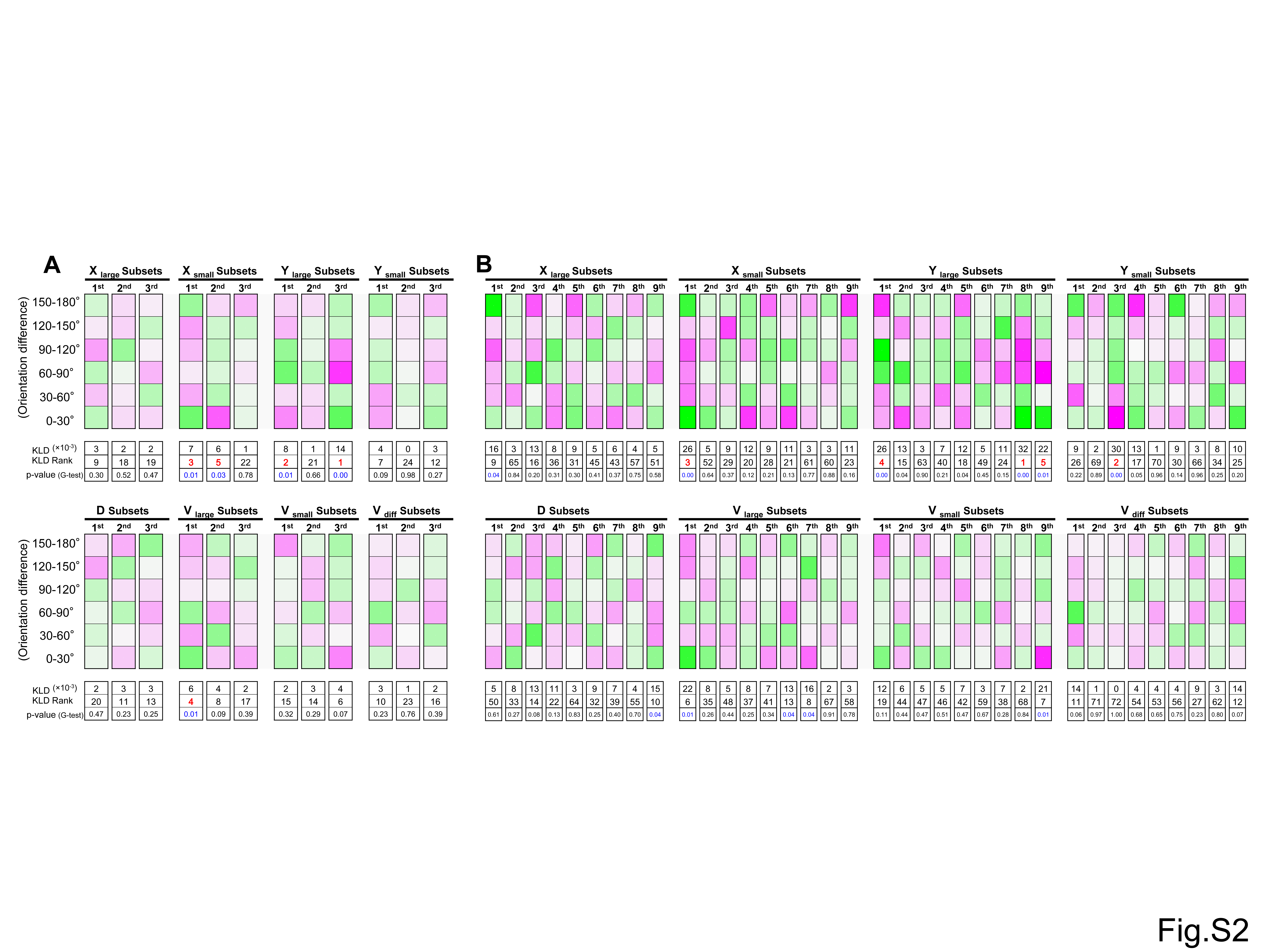

Supplement: Figure S2 — KLD and p value based on other subdivisions (3 and 9). We represent the probability distribution using a heat map. The OD histogram-intervals with a higher proportion than that of all combinations are shown in green, while those with a lower proportion are shown in magenta. The KLD value and KLD rank are indicated below each column. Below them, p values for the G-test are shown. (a) 3 subsets division based 8 explanatory variables. (b) 9 subsets division based 8 explanatory variables. (TIF) [file pone.0071685.s002.tif]

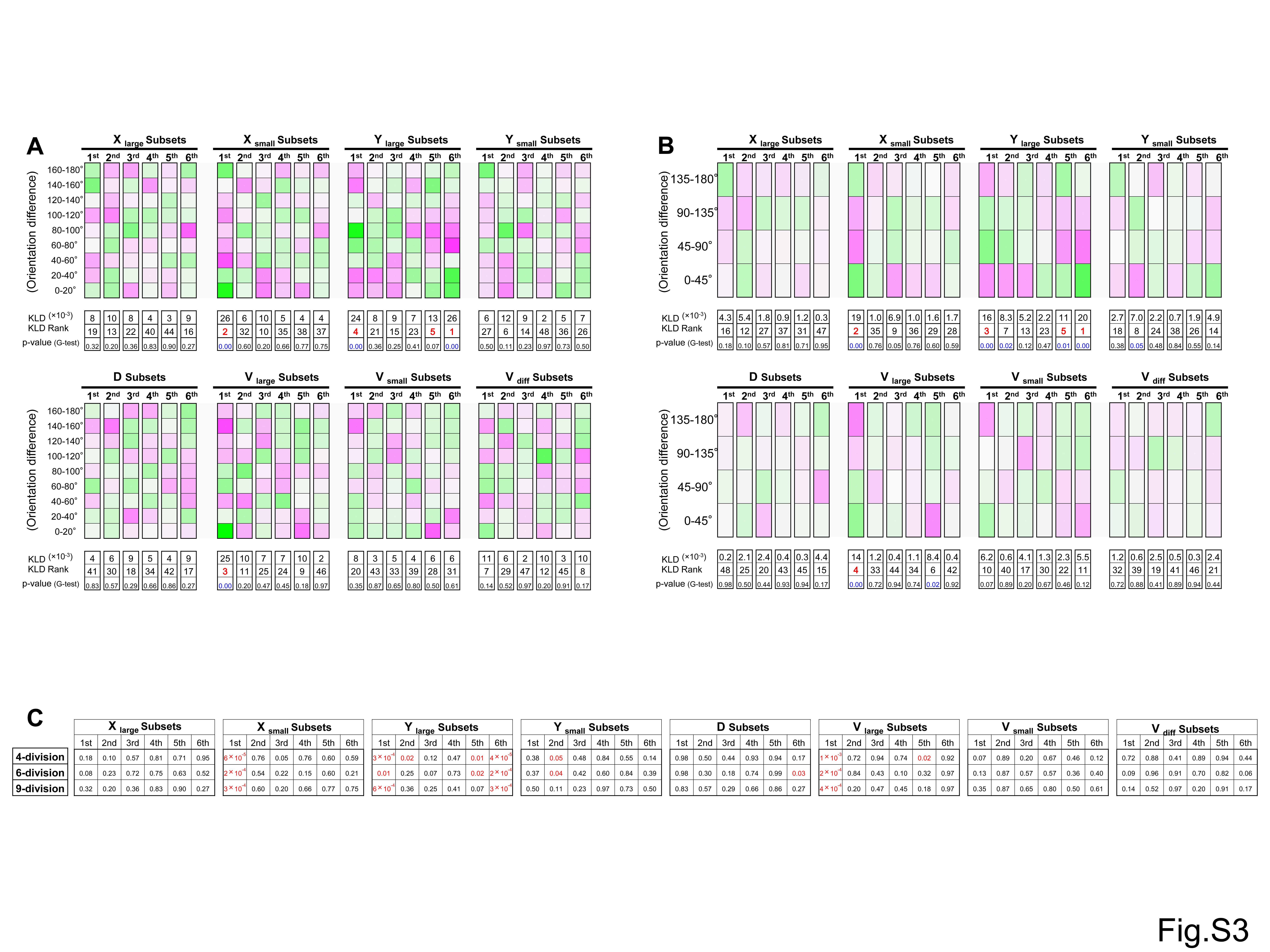

Supplement: Figure S3 — KLD and p value when the discrete probability distribution has 4 or 9 intervals. (A) 9-intervals. We represent the probability distribution using a heat map. The OD distribution has 9 intervals; [0°–20°], [20°–40°], [40°–60°], [60°–80°], [80°–100°], [100°–120°], [120°–140°], [140°–160°], and [160°–180°]. The OD histogram-intervals with a higher proportion than that of all combinations are shown in green, while those with a lower proportion are shown in magenta. The KLD value and KLD rank are indicated below each column. Below them, p values for the G-test are shown. (B) 4-intervals. The probability distribution has 4 intervals; [0°–45°], [45°–90°], [90°–135°], and [135°–180°]. (C) Comparison of p value on G-test. The p values under 0.05 are colored blue. (TIF) [file pone.0071685.s003.tif]

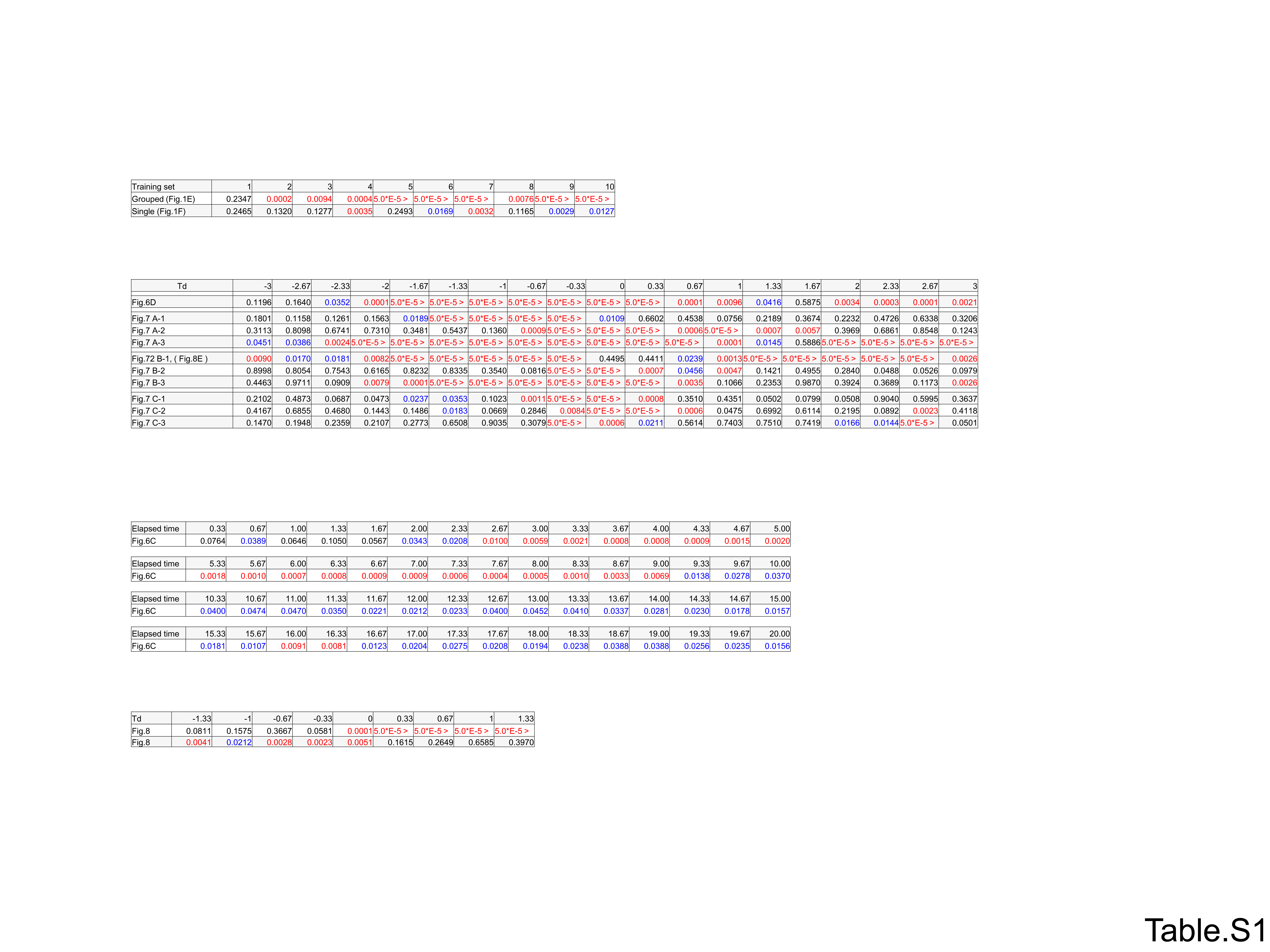

Supplement: Table S1 — Summary for p values in Figs. 1 , 6 , 7 , and 8 . A p value less than 0.01 is indicated in red, and that less than 0.05 is indicated in blue. (TIF) [file pone.0071685.s004.tif]
